# A Two-Cohort RNA-seq Study Reveals Changes in Endometrial and Blood miRNome in Fertile and Infertile Women

**DOI:** 10.3390/genes9120574

**Published:** 2018-11-23

**Authors:** Kadri Rekker, Signe Altmäe, Marina Suhorutshenko, Maire Peters, Juan F. Martinez-Blanch, Francisco M. Codoñer, Felipe Vilella, Carlos Simón, Andres Salumets, Agne Velthut-Meikas

**Affiliations:** 1 Institute of Clinical Medicine, Department of Obstetrics and Gynecology, University of Tartu, 50406 Tartu, Estonia; marina.suhorutsenko@ut.ee (M.S.); maire.peters@ut.ee (M.P.); andres.salumets@ccht.ee (A.S.); 2 Competence Centre on Health Technologies, 50410 Tartu, Estonia; signealtmae@ugr.es (S.A.); agnevelthut@gmail.com (A.V.-M.); 3 Department of Biochemistry and Molecular Biology, Faculty of Sciences, University of Granada, 18071 Granada, Spain; 4 Lifesequencing SL, 46980 Valencia, Spain; juan.martinez@lifesequencing.com (J.F.M.-B.); francisco.codoner@adm.com (F.M.C.); 5 Igenomix Foundation/INCLIVA, 46010 Valencia, Spain; felipe.vilella@igenomix.com (F.V.); carlos.simon@igenomix.com (C.S.); 6 Igenomix SL, 46980 Valencia, Spain; 7 Department of Obstetrics and Gynecology, Valencia University, 46010 Valencia, Spain; 8 Institute of Biomedicine and Translational Medicine, Department of Biomedicine, University of Tartu, 50412 Tartu, Estonia; 9 Department of Obstetrics and Gynecology, University of Helsinki and Helsinki University Hospital, FI-00014 Helsinki, Finland; 10 Department of Chemistry and Biotechnology, Tallinn University of Technology, 12618 Tallinn, Estonia

**Keywords:** endometrial receptivity, infertility, microRNA, recurrent implantation failure, small RNA-seq

## Abstract

The endometrium undergoes extensive changes to prepare for embryo implantation and microRNAs (miRNAs) have been described as playing a significant role in the regulation of endometrial receptivity. However, there is no consensus about the miRNAs involved in mid-secretory endometrial functions. We analysed the complete endometrial miRNome from early secretory (pre-receptive) and mid-secretory (receptive) phases from fertile women and from patients with recurrent implantation failure (RIF) to reveal differentially expressed (DE) miRNAs in the mid-secretory endometrium. Furthermore, we investigated whether the overall changes during early to mid-secretory phase transition and with RIF condition could be reflected in blood miRNA profiles. In total, 116 endometrial and 114 matched blood samples collected from two different population cohorts were subjected to small RNA sequencing. Among fertile women, 91 DE miRNAs were identified in the mid-secretory vs. early secretory endometrium, while no differences were found in the corresponding blood samples. The comparison of mid-secretory phase samples between fertile and infertile women revealed 21 DE miRNAs from the endometrium and one from blood samples. Among discovered novel miRNAs, chr2_4401 was validated and showed up-regulation in the mid-secretory endometrium. Besides novel findings, we confirmed the involvement of miR-30 and miR-200 family members in mid-secretory endometrial functions.

## 1. Introduction

The establishment of a receptive endometrium is essential for successful embryo implantation. The endometrium is receptive within a limited time frame during the mid-secretory cycle phase and aberrations in the processes involved in transition to this stage can lead to infertility [1,2]. Indeed, impaired endometrial receptivity is suspected to play a major role in female infertility among women suffering from recurrent implantation failure (RIF). Gaining insights into the complex mechanisms controlling changes within the endometrium is crucial to understanding not only embryo implantation but also endometrial dysfunction that can lead to infertility. Although hundreds of simultaneously up- and down-regulated genes have been implicated in the processes of acquiring endometrial receptivity [3,4] and the development of RIF [5,6,7,8,9,10], the precise molecular mechanisms regulating gene expression necessary for endometrial function in fertility and infertility-associated diseases are not well understood.

MicroRNAs (miRNAs), non-coding RNAs (ncRNAs) of ~22 nucleotides in length, act as post-transcriptional regulators of gene expression by inhibiting the stability or repressing the translation of their target messenger RNA (mRNA) molecules [11]. To date, more than 2500 annotated miRNAs in the human genome are known, and as each miRNA may regulate hundreds of genes, it is estimated that miRNAs collectively adjust the expression of over one third of genes in the human genome [11,12]. Hence, miRNAs orchestrate a large variety of processes, including the cyclic changes in the female reproductive tract [13] and cellular processes involved in implantation, such as cellular differentiation, proliferation and apoptosis [14].

The involvement of miRNAs in the mid-secretory endometrial functions has been demonstrated [15,16,17] and aberrant miRNA profiles in RIF have been identified in a few previous microarray-based [18,19,20,21] and two RNA-sequencing (RNA-seq) based studies [22,23]. However, as the analysis settings and platforms have been different and only a small number of samples have been analysed, there is no consensus about the list of miRNAs involved in endometrial receptivity and in endometrial dysfunction(s), advocating further investigations.

Therefore, we set out to analyse the complete spectrum of endometrial miRNAs–miRNome from paired early secretory (ES) and mid-secretory (MS) phase samples collected from two independent population cohorts using the comprehensive RNA-seq technology. Additionally, MS endometrial samples from RIF patients were included in order to identify the dysregulated miRNAs in infertility. Furthermore, we investigated whether the overall cyclic changes in the female body during ES to MS transition and with RIF condition could also be reflected in blood miRNA profiles.

## 2. Materials and Methods

### 2.1. Study Participants

The study was approved by the Research Ethics Committee of the University of Tartu, Estonia (No 221/M-31), and the Ethical Clinical Research Committee of IVI Clinic, Valencia, Spain (No 1201-C-094-CS). Informed consent was signed by all women who entered the study and all research was performed in accordance with relevant guidelines and regulations. 

The study participants were recruited and samples were collected by two independent research teams from two countries: Estonia (EST) and Spain (ESP). All protocols were standardized across the study centres with exceptions highlighted in the “Sample collection” paragraph.

The healthy fertile group consisted in total of 39 women: EST *n* = 22, age 30.2 ± 3.3 years (mean ± standard deviation), body mass index (BMI) 23.1 ± 4.2 kg/m^2^; and ESP *n* = 17, age 29.4 ± 3.6 years, BMI 23.2 ± 2.8 kg/m^2^; with self-reported regular menstrual cycles. All women had at least one live-born child (1.5 ± 1.0 for EST cohort; 1.2 ± 0.4 for ESP cohort) within the last 10 years (5.3 ± 2.6 years, data available for the EST cohort) and no previous infertility records.

RIF patient group consisted in total of 38 women: EST *n* = 21, age 35.1 ± 3.9 years, BMI 22.3 ± 2.3 kg/m^2^; ESP *n* = 17, age 36.7 ± 3.3 years, BMI 25.2 ± 5.3 kg/m^2^. All RIF patients had undergone at least three (4.0 ± 1.5 for EST cohort; 3.0 ± 1.7 for ESP cohort) unsuccessful in vitro fertilisation (IVF) treatment cycles with embryo transfers. No donor eggs were used in the treatments. RIF patients were not undergoing hormonal stimulation for IVF at the time of sample collection. Among these patients, tubal and male factor were the main reasons for infertility along with endometriosis and unexplained infertility. Two participants were suffering from secondary infertility with the experience of childbirth in average 6.5 ± 0.5 years ago. None of the participants had received hormonal treatments for at least three months prior to the time of sample collection.

### 2.2. Sample Collection

All women performed urine-based ovulation tests to determine the luteinizing hormone (LH) surge using commercial kits (BabyTime^®^ hLH urine cassette, Pharmanova, Beit Shemesh, Israel). The day with positive outcome of the ovulation test is referred to as LH + 0. Healthy women donated endometrium and blood samples during two time-points of the same menstrual cycle: LH + 1 to LH + 3 corresponding to the ES phase and LH + 7 to LH + 9 corresponding to the MS phase. Women in the RIF group provided samples at MS phase (LH + 7 to LH + 9).

Endometrial tissue was obtained using Pipelle catheter (Laboratoire CCD, Paris, France). The biopsy was divided into two parts: one part was rinsed in sterile phosphate-buffered saline (PBS) and frozen at −80 °C in RNA*later* solution (Thermo Fisher Scientific, Waltham, MA, USA) in EST or without any additives in ESP, the other portion was placed in 10% buffered formalin for histological evaluation. Histological dating was conducted to confirm the endometrial phase. In addition, the receptivity status of all endometrial biopsies was assessed and confirmed by analysing a set of 57 receptivity biomarkers as part of our previous studies [4,24].

Blood samples were collected and processed differently by the two research centres. In EST, whole blood samples were collected into PAXgene Blood RNA Tubes (Qiagen, Hilden, Germany), incubated at room temperature for 2 h and then frozen until further use. In ESP, blood samples were collected with tubes containing K_2_-EDTA anticoagulant, the buffy coat fractions containing leukocytes and platelets were separated by a centrifugation procedure with Histopaque-1077 (Sigma-Aldrich, St. Louis, MO, USA), and were frozen until further use.

### 2.3. RNA Extraction from Endometrial Tissue

Up to 30 mg of the endometrial tissue was processed using miRNeasy Mini and RNeasy MinElute kits (Qiagen), following the manufacturer’s protocol for isolating small RNA (<200 nucleotides) separately from large RNA molecules. DNase I treatment was performed on column using RNase-Free DNase Set (Qiagen). Purified RNA quantity was determined with Bioanalyzer 2100 Small RNA kit (Agilent Technologies, Santa Clara, CA, USA).

### 2.4. RNA Extraction from Blood Samples

In EST, RNA was extracted from the whole blood samples using PAXgene Blood miRNA Kit (Qiagen) according to manufacturer’s instructions. In ESP, total RNA was isolated from buffy coat (leukocytes and platelets) using the miRNeasy Mini Kit and then cleaned with RNA Cleanup kit (Qiagen). On-column DNase I treatment was performed with RNase-Free DNase Set (Qiagen) for all samples.

### 2.5. Small RNA Sequencing

**Figure 1:**
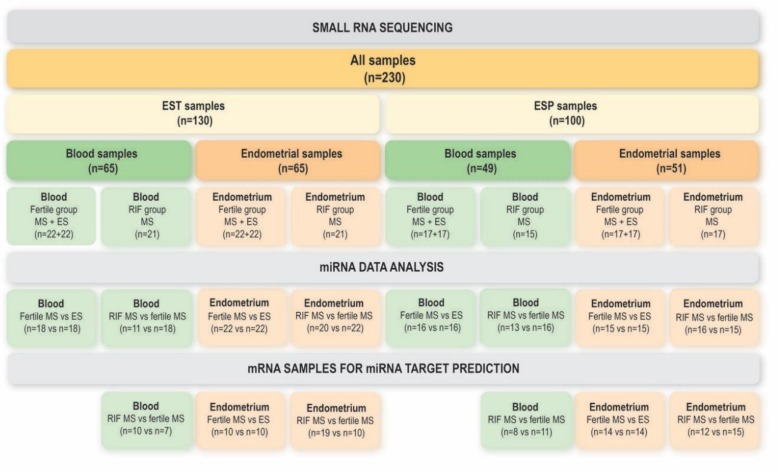
Number of samples included to small RNA sequencing, to final microRNA (miRNA) analysis and to messenger RNA (mRNA) sequencing analysis. EST—Estonian samples; ESP—Spanish samples; RIF—recurrent implantation failure; ES—early secretory phase; MS—mid-secretory phase.

A total of 116 endometrial samples (EST *n* = 65; ESP *n* = 51) and 114 blood samples (EST *n* = 65; ESP *n* = 49) were subjected to small RNA sequencing in the current study (Figure 1). Small RNA libraries were constructed following the TruSeq Small RNA Library Preparation Guide (Illumina, San Diego, CA, USA). As input, 1 µg of total RNA or small RNA fraction was used; 12-plexing of samples was performed by inserting Illumina index sequences into each library. Final purification step was performed by manually selecting libraries corresponding to the length of inserted miRNAs (area between 145–160 bp) using gel electrophoresis (6% Novex TBE gels). Libraries were quantified and validated with Agilent 2100 Bioanalyzer (Agilent Technologies), normalized, pooled, used for cluster generation on the cBot and sequenced on HiSeq 2000/2500 with a configuration of 50 cycles Single Reads following manufacturer’s instructions (Illumina) at Lifesequencing S.L., Valencia, Spain (ESP samples) or at Estonian Genome Center Core Facility, Tartu, Estonia (EST samples).

### 2.6. SMALL RNA Sequencing Data Analysis

Small RNA sequencing data were deposited into the Gene Expression Omnibus (GEO accession number GSE108966). Quality control and adapter trimming of raw sequences was performed using Trimmomatic version 0.36 [25]. All sequences were scanned with 4-base long sliding window and reads were dropped if the average quality of base pair was below 30 according to the Phred +33 quality score system or if total read length was less than 17.

Identification of miRNAs from sequencing reads was performed by the miRDeep2 algorithm [26]. Firstly, mapper.pl algorithm was used to map the trimmed high-quality sequences to the UCSC human genome version hg19. Common reads were collapsed and passed as input to miRDeep2.pl algorithm together with mature and stem-loop sequences of human miRNAs from miRBase version 21 [27]. Standard miRDeep2 settings were used for the analysis.

Raw read counts for each miRNA from miRDeep2 analysis were used as input for differential expression analysis in edgeR package version 3.16.5 [28,29]. Analysis was run on R software environment version 3.3.1. Samples with library size below 500,000 reads and those performing as clear outliers according to the multidimensional scaling (MDS) plot were omitted. The remaining number of patient samples used for each comparison is displayed in Figure 1. Count per million (CPM) of at least 1 in 75% of samples in the smaller group was set as a threshold for each miRNA to be included in the analysis for each separate comparison. The remaining raw library sizes were scaled according to the method of weighted trimmed mean of M-values [30].

For comparing samples from ES and MS phases, a generalized linear model (GLM) likelihood ratio test with paired design was performed. For comparison of samples from fertile group and from patients with RIF, GLM test with age adjustment was implemented as age between those two groups was statistically different (*t*-test, *p* < 0.05). EST and ESP datasets were analysed separately and miRNAs that were detected as differentially expressed (DE) in both sample sets were included for further analysis.

### 2.7. Novel miRNA Identification

Novel miRNAs were predicted from 92 blood samples and 110 endometrial samples (Table 1). The reads predicted as potential candidate miRNAs by miRDeep2 were subjected to BLAST in order to discriminate the sequences corresponding to other human ncRNAs such as rRNA, snRNA, tRNA, lncRNA etc. Mature miRNA sequences of *Pan troglodytes* were included as input for evolutionary conservation analysis, which provides more solid confirmation for finding novel unannotated miRNAs from high-throughput sequencing experiments [31]. Sequences were considered as potentially novel miRNAs if they met the following criteria: (a) miRDeep2 score ≥ 5, (b) significant randfold *p*-value, (c) detected in at least 3 samples within a sample/biofluid type (endometrium or blood), (d) detected from two different datasets (EST + ESP) or detected from different sample types (endometrium and blood). miRDB was used for novel miRNA target prediction [32] and Ingenuity Pathway Analysis (IPA, 2017, Qiagen Bioinformatics Redwood City, CA, USA) was performed on predicted targets.

### 2.8. mRNA Data Analysis for miRNA Target Prediction

For miRNA target gene prediction purposes, mRNA data from our previously published RNA-seq study were retrieved from Gene Expression Omnibus (GSE98386) [4,24]. The mRNA dataset comprised of a subset of the same samples that were used for miRNA analysis in the current study (Figure 1). Raw mRNA sequencing read quality was inspected with FastQC v.0.11.3 (http://www.bioinformatics.babraham.ac.uk/projects/fastqc/) before and after the preprocessing step. Adapter removal and trimming was performed with Trimmomatic tool v. 0.32 [25]. Quality threshold was set to 30 according to Phred-33 scoring system and reads <36 nucleotides were omitted. FASTQ Quality Filter from FASTX-Toolkit v. 0.0.13 (http://hannonlab.cshl.edu/fastx_toolkit/index.html) was used as a second level of quality control, where the average quality threshold of 30 for every 50 nucleotides was set. Good quality reads were mapped to the human reference genome hg19 using TopHat v. 2.0.11 [33]. Read counts for every gene in Ensembl v.75 annotation file were generated with HTSeq v.0.6.1 [34]. Differential gene expression analysis was performed by using edgeR package according to the same parameters as described in miRNA data analysis. List of DE mRNAs that were common for EST and ESP samples were passed to miRNA-mRNA interaction analysis.

### 2.9. Integrated Analysis of miRNA and mRNA Data

Differentially expressed miRNAs and mRNAs in endometrial samples from ES vs. MS phases from fertile women and from MS phase of RIF patients were further analysed using the software IPA. The tool microRNA Target Filter was applied which allows prioritization of experimentally validated and predicted mRNA targets from TargetScan, TarBase, miRecords, and the Ingenuity Knowledge Base. Expression pairing of our miRNA and mRNA RNA-seq data was performed using the confidence filter of ‘experimentally observed’ and ‘high confidence’, and the expression pairing filter of opposite expression directions was applied (pairs of up-regulated miRNA with down-regulated mRNA, and down-regulated miRNA with up-regulated mRNA). The involvement of detected miRNA target genes in different canonical pathways was analysed. The overall level of gene expression in the canonical pathways was analysed by GOplot package version 1.0.2 in R statistical environment. The z-score refers to the level of under- or overrepresented mRNAs in each pathway and is calculated as z = (number of up-regulated genes − number of down-regulated genes)/√count [35].

**Figure 2:**
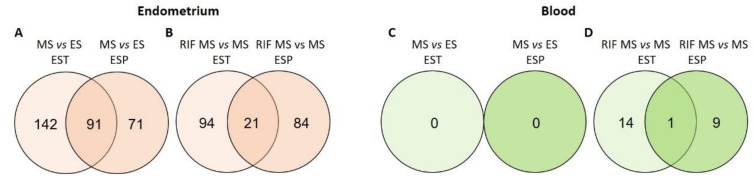
Number of differentially expressed miRNAs in EST and validation cohort ESP in (**A**) MS vs. ES phase endometrial samples of fertile women; (**B**) MS phase endometrial samples from infertile recurrent implantation failure (RIF) patient vs. fertile women; (**C**) MS vs. ES phase blood samples of fertile women; and (**D**) MS phase blood samples from RIF patients vs. fertile women. EST - Estonian samples; ESP—Spanish samples; RIF—recurrent implantation failure; ES—early secretory phase; MS—mid-secretory phase.

miRNA–mRNA interactions were not analysed on data from blood samples because no DE miRNAs between blood samples corresponding to MS vs. ES phase were identified in either of the two cohorts of fertile women (Figure 2), and no consensus mRNAs were identified between EST and ESP cohorts when comparing blood mRNAs from MS cycle phase of fertile vs. RIF women.

### 2.10. Novel miRNA Validation Using Quantitative Real-Time Polymerase Chain Reaction

Custom TaqMan Small RNA assay (Thermo Fisher Scientific) was used for novel miRNA (chr2_4401, sequence: gaacacugaaguuaauggcug) validation with eight paired ES and MS endometrial samples from fertile women and eight MS endometrial samples from RIF women. The average level of miR-151a-5p and miR-196b-5p was used as reference for normalization. These reference miRNAs were chosen due to their stable expression levels according to the current small RNA sequencing data. Additionally, five paired endometrial stromal (CD13^+^) and epithelial cell (CD9^+^) samples (two from ES and three from MS phase) isolated by fluorescence-activated cell sorting (FACS) were used to determine the cell type specificity of the novel miRNA. Cell sorting was performed according to our previous publication [36]. cDNA synthesis was conducted with TaqMan MicroRNA Reverse Transcription Kit (Thermo Fisher Scientific) and quantitative real-time polymerase chain reaction (qRT-PCR) was performed with TaqMan Universal PCR Master Mix, No AmpErase UNG (Thermo Fisher Scientific). Real-time experiments were performed in duplicate. Relative miRNA expression levels were compared between the studied groups by paired (ES vs. MS) or unpaired (fertile vs. RIF) two-tailed *t*-test (Excel, Microsoft Corporation, Redmond, WA, USA) and *p*-value ≤ 0.05 was considered as significant. Fold change (FC) was calculated using the 2^−ΔΔCt^ method [37].

### 2.11. Data Availability

The datasets analysed during the current study are available in the Gene Expression Omnibus repository (GEO), accession numbers GSE108966 and GSE98386.

## 3. Results

### 3.1. miRNAs in the Endometrium

In this study, 65 endometrial samples from the Estonian cohort (EST) and 51 samples from independent Spanish validation cohort (ESP) were included for miRNA data analysis (Figure 1). In total, 615 miRNAs were detected from endometrial samples among the EST cohort and 624 miRNAs among ESP cohort (CPM of at least 1 in 75% of samples within a sample group of ES or MS of fertile group, or RIF). Most abundant miRNAs in endometrial tissues from both cohorts were miR-10b-5p, miR-10a-5p and miR-27b-3p (Table S1A,B).

miRNAs were considered as DE if they showed significantly altered levels (FDR < 0.05) also in validation cohort (both in EST and ESP). From fertile women, 91 DE endometrial miRNAs were confirmed from MS vs. ES (Figure 2A) of which 49 were down- and 42 up-regulated in MS endometrium (Table S2A).

The comparison of MS endometrial samples from RIF patients vs. fertile women in EST and ESP validation datasets revealed 21 DE miRNAs (Figure 2B), out of which eight miRNAs were more abundantly expressed in RIF patients and 13 miRNAs in fertile women (Table S2B).

miR-424-5p was the only DE miRNA present in both comparisons—between the ES and MS endometria of fertile women (Table S2A) as well as between fertile women and RIF patients (Table S2B). Interestingly, while down-regulated in the MS endometrium of fertile women (average FC = −1.87 between the two cohorts), it was up-regulated in RIF patients’ samples from the same phase (average FC = 1.74). When predicting miR-424-5p target genes from our mRNA dataset (Figure 1, see Methods section), we detected 85 targets (Table S3A) with involvement in different canonical pathways important in MS endometrial functions such as glucocorticoid, insulin receptor, axonal guidance and interleukins signalling (Table S4A). The miRNA-mRNA target analysis among endometrial samples from RIF women, based on our experimental datasets, identified miR-424-5p to target solely Serine/Threonine Kinase 2 (*SGK2*) gene (Table S3B).

### 3.2. miRNAs in Blood

Altogether, 65 blood samples from EST and 49 samples from ESP validation cohort were included for miRNA data analysis (Figure 1). In total, 305 miRNAs were detected from blood samples among EST cohort and 710 miRNAs among the independent ESP cohort (Table S1C,D). miRNAs were isolated from whole blood in EST samples and from the buffy coat fraction in ESP samples. Possibly due to the differences in sample treatment protocols between the centres, the most abundant miRNAs in blood varied between the two cohorts: miR-486-5p, miR-92a-3p and miR-451a demonstrated the highest read counts among EST blood samples, while miR-26a-5p, miR-191-5p and miR-181a-5p exhibited the highest expression levels among ESP samples.

No DE miRNAs were detected between blood samples corresponding to ES and MS cycle phase in fertile women either in the EST dataset or in the validation ESP dataset (Figure 2C). The comparison of blood samples corresponding to MS cycle phase from fertile and RIF women revealed that miR-30a-5p was significantly up-regulated among RIF patients in EST cohort (FC = 3.0, FDR = 0.01); and the difference was also confirmed in ESP cohort (FC = 1.9, FDR = 0.03) (Figure 2D).

### 3.3. Novel miRNAs

In total, 18 novel miRNAs were determined from endometrial and blood samples (Table 1, Table S5). Out of these miRNAs, chr2_1900 (sequence: aucugaaauuugaaauggucc) and chr16_22077 (aggcuaggcugggccacag) were detected only from MS endometrial samples (from 7 and 4 out of 73 MS samples, respectively) and chr2_4401 (gaacacugaaguuaauggcug) was found from the majority (75.3%; 55/73) of MS endometrial samples and only from 2.7% (1/37) of ES endometrial samples. chr14_10307 (ucugagcccuguucucccuagg) was uniquely determined from blood samples of 16.7% RIF patients (4 out of 24 RIF blood samples).

**Figure 3:**
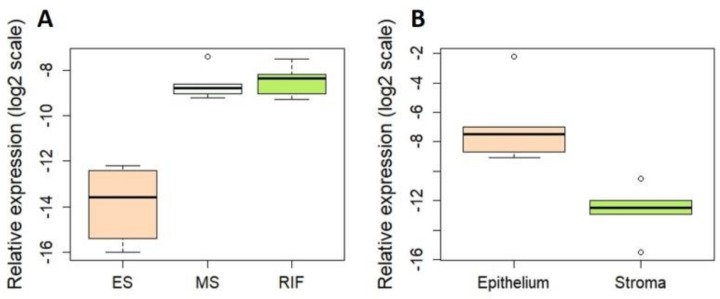
Validation of the novel miRNA chr2_4401 by quantitative real-time polymerase chain reaction (qRT-PCR). (**A**) miRNA expression level differences between paired ES (*n* = 8) and MS (*n* = 8) endometria of fertile women, and MS endometria of RIF (*n* = 8) patients. The relative miRNA expression levels (ΔCt) in the endometrium were 37-fold higher in MS compared to ES samples (*p* = 0.0001). No differences between MS samples of fertile and RIF patients were observed. (**B**) Relative expression level differences in epithelial (*n* = 5) and stromal (*n* = 5) fractions. The relative miRNA levels (ΔCt) were 55-fold higher in epithelial cells (CD9+) compared to stromal cells (CD13+) (*p* = 0.003). The average levels of miR-151a-5p and miR-196b-5p were used for data normalization. For illustrative purposes, relative expression levels (ΔCt) were multiplied by −1. ES—early secretory phase; MS—mid-secretory phase.

We further focused on the most promising novel miRNA chr2_4401. Validation analysis by qRT-PCR confirmed the differential expression of chr2_4401 showing that the level of this novel miRNA was 37-fold higher (*p* = 0.0001) in MS compared to ES endometrium in fertile women (Figure 3A). No statistically significant differences in the expression levels of chr2_4401 between the MS endometrial samples from fertile and RIF women were detected (Figure 3A). Cell type-specific expression analysis showed very low levels of chr2_4401 in endometrial stromal cells, but 55-fold upregulation in epithelial cells was detected (*p* = 0.003, Figure 3B).

**Figure 4:**
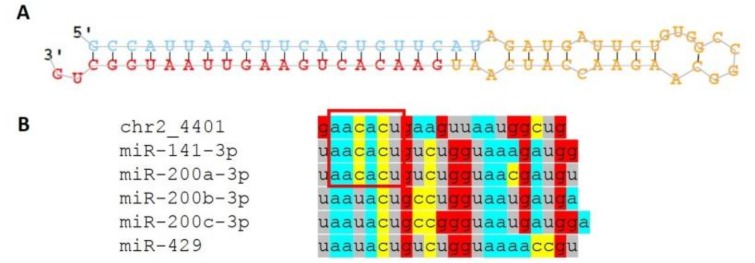
(**A**) Hairpin structure of the novel miRNA chr2_4401 precursor predicted by miRDeep2. (**B**) Sequence alignment between chr2_4401 and five miR-200 family miRNAs. The seed region (nucleotides 2–7, red square) of chr2_4401 is identical with miR-141-3p and miR-200a-3p.

The novel miRNA chr2_4401 precursor sequence (Figure 4A) is located within the short arm of chromosome 2 (p11.2) and is transcribed from intergenic region. No human miRNAs with high similarity sequences for the predicted novel miRNA was found from miRBase database, however, chr2_4401 shares the seed region (2–7 nt) with miR-200 family miRNAs (Figure 4B) and, therefore, target genes of the predicted miRNA are expected to be common with those of the miR-200 family. miRDB revealed more than 800 potential target genes for chr2_4401 (Table S6A). For pathway analysis, we focussed only on these potential targets that were detected as down-regulated in the MS endometrium in our mRNA-seq analysis from the same women (98 genes, Table S6B). The predicted target genes of this novel miRNA identified in our dataset were involved in pathways including estrogen-mediated S-phase entry (*p* = 0.006), cell cycle regulation by B-cell translocation gene (BTG) family proteins (*p* = 0.01), role of checkpoint kinase (CHK) proteins in cell cycle checkpoint control (*p* = 0.03), and epithelial adherens junction signalling (*p* = 0.03) (Table S4B).

### 3.4. miRNA–mRNA Interaction Prediction

**Figure 5:**
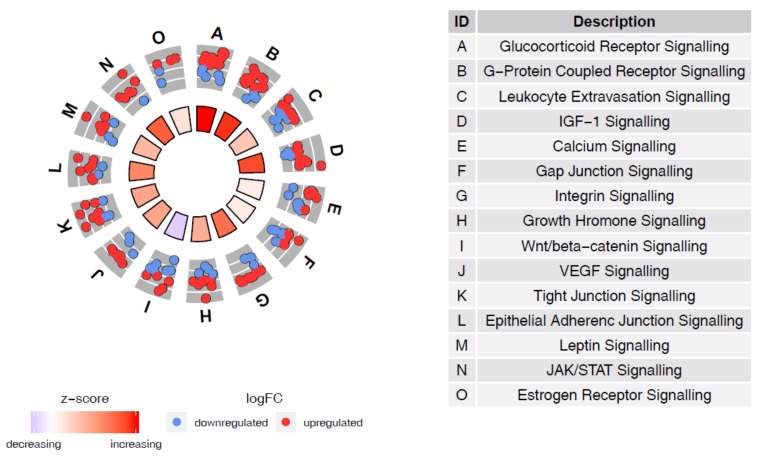
Circular plot of IPA canonical pathways enriched with miRNA targets differentially expressed between MS and ES endometrial samples of fertile women. The inner circle represents the overall up- or down-regulation of gene expression in each pathway according to the z-score. The outer circle depicts the fold change of expression level for each gene.

The joint analysis of the miRNA and mRNA RNA-seq data from MS vs. ES endometrial samples by IPA software detected 81 (out of 91) DE miRNAs and 865 (out of 4240) mRNAs (Table S3A). Further analysis demonstrated that these 865 target mRNAs are involved in canonical pathways important in several MS endometrial functions (Table S4C). According to the calculated z-scores, differential miRNA expression leads to the up-regulation of gene expression in the glucocorticoid, G-protein coupled receptor, IGF-1 and JAK/STAT signalling pathways (obtaining the highest z-score), while the most significant down-regulation of gene expression was observed within Wnt/beta-catenin signalling pathway (as demonstrated by the lowest z-score) (Figure 5).

The analysis of our miRNA and mRNA sequencing data from MS endometrial samples from fertile vs. RIF women detected 21 DE miRNAs and 14 mRNAs. Out of those the IPA program identified 4 miRNAs in interaction with 5 mRNAs (Table S3B). The miRNA targets were detected to be involved in signal transducer and activator of transcription 3 (STAT3) and cyclin-dependent kinase 5 (CDK5) signalling pathways (Table S4D).

## 4. Discussion

Current knowledge about the involvement of miRNAs in endometrial transformation from ES (pre-receptive) to MS (receptive) stage both in fertility as well as in infertility conditions is limited. Moreover, it is not known whether the overall systemic changes in women during these processes are also reflected in blood miRNA profiles, which could serve as non-invasive biomarker candidates for evaluating fertility status. Therefore, using small RNA-seq technology, we profiled and validated the complete endometrial and blood miRNome from ES and MS phase samples from healthy fertile women and infertile RIF patients in two independent sample cohorts. This is the largest miRNA endometrial receptivity study to date with novel aspects of combining matched samples of the endometrium and blood from two different populations.

We identified 91 DE miRNAs between the ES and MS endometrium of healthy fertile individuals, showing the same directions in miRNA expression in both cohorts. Several miRNAs that have repeatedly been associated with endometrial receptivity were also confirmed by our study, including miR-30b-5p, miR-30d-3p, miR-30d-5p and miR-30a-5p that were up-regulated in the MS phase endometrium. Endometrial expression level changes of the miR-30 family members have been described in a number of previous endometrial receptivity studies [4,15,16,17,22,23,38]. Furthermore, it has been previously shown that endometrial miR-30d is taken up by the pre-implantation embryo, resulting in modified transcriptome and embryo adhesion [16]. Therefore, our data together with previous studies collectively indicate that the miR-30 family is crucial in the regulation of endometrial receptivity and implantation processes in the endometrium.

Another interesting group of miRNAs in endometrial receptivity is the miR-200 family. We detected several miR-200 family members, such as miR-200a-3p, miR-200c-3p, miR-200c-5p, miR-141-3p, miR-141-5p and miR-429 to be up-regulated in the MS phase endometrium, being in line with previously published studies [16,23]. miR-200 family members are shown to target several genes in the endometrium that influence cell proliferation, migration and inflammation [39], which are all important cellular processes in MS endometrial functions. Interestingly, the miR-200 family is predominantly expressed in endometrial epithelial cells [36], which is the first cellular layer to interact with the implanting embryo. Nevertheless, as whole tissue biopsies were analysed in our study, we cannot confirm that the detected findings are epithelial cell specific.

Our study results also identified miRNAs that, to the best of our knowledge, have not been associated with human endometrial receptivity before (Table S2A). For instance, miR-873-3p was one of the most up-regulated and miR-3131 one of the most down-regulated miRNAs in the MS phase endometrium from fertile women. The roles of these miRNAs in endometrial tissue remain to be elucidated, but it has been demonstrated that miR-873-3p plays a role in the selection of bovine dominant follicle [40].

Interaction analysis of the DE miRNAs and their target genes identified the involvement of several canonical pathways important in endometrial receptivity, including JAK/STAT signalling, leptin signalling and growth hormone signalling. JAK/STAT signalling pathway transmits information from extracellular signals to the nucleus influencing transcription, and its involvement in embryo implantation is widely acknowledged [41,42,43,44]. Leptin signalling, regulated by cytokines and playing a role in inflammatory response, is another classically known signalling pathway to be involved in endometrial receptivity [43,45]. Furthermore, leptin is known to mediate the effects of growth hormone [46]. The beneficial effects of growth hormone administration on endometrial receptivity have recently been published, where growth hormone increased endometrial blood perfusion and the expression of different cytokines [47]. Growth hormone administration has also improved implantation, pregnancy and live birth rates in RIF patients [48]. The Wnt/beta-catenin signalling pathway has been demonstrated to control estrogen-dependent endometrial cell proliferation, decidualisation, trophoblast attachment and invasion. Fine-tuning of this pathway is particularly important as failures in Wnt signalling are associated with infertility, endometriosis, endometrial cancer and gestational diseases such as complete mole placentae and choriocarcinomas (reviewed in [49]). miRNAs differentially expressed upon the establishment of receptivity participate in the overall down-regulation of Wnt/beta-catenin signalling in the normal endometrium according to our results.

The endometrial biopsies from healthy participants were all taken at two time-points (ES and MS) within the same menstrual cycle. A recent study by Evans et al. demonstrated that the prior collection of an LH + 2 sample does not affect the general gene expression level of a LH + 7 sample [50]. Their study data suggested that the expression of tested genes exhibited a stable pattern, which was not affected even when sampled twice in one cycle and is valid to predict endometrial receptivity in the subsequent cycles. No such studies have been performed regarding miRNA expression, but similar variability in expression levels is expected within and between cycles as for mRNAs.

Besides the considerable changes in endometrial tissue, cyclic alterations take place throughout the female body during the menstrual cycle under the governance of steroid hormones. In order to find systemic miRNA changes as indirect markers that could reflect the receptive stage of the endometrium, we analysed blood samples that were collected simultaneously to endometrial biopsies. However, no DE blood miRNAs were found between the samples corresponding to ES and MS time-points in either of the cohorts, regardless of the sample isolation protocol. Prior to our current study, miRNAs from whole blood have not been investigated in abovementioned time-points, but we have previously shown that there are no differences in circulating plasma miRNA profile throughout the menstrual cycle [51]. Therefore, it is probable that there are no blood-derived miRNAs that could reflect the changes which occur in female body during the establishment of the receptive endometrium.

Another interesting aspect of our study was the identification of dysregulated miRNAs among RIF women. Although this study group comprised of patients with various causes of infertility, they all had in common at least three unsuccessful IVF treatments with embryo transfers. We identified 21 DE miRNAs in the MS endometrium of RIF patients compared to fertile women. Notably, as has also been previously reported [21,22,23], miR-424-5p was up-regulated in RIF patients, while being down-regulated in the mid-secretory endometrium of healthy fertile women. Therefore, miR-424-5p may be a useful marker to determine endometrial insufficiency for embryo implantation. miR-424-5p has been previously shown to stimulate cell-to-cell adhesion during embryo implantation by targeting osteopontin (encoded by *SPP1* gene) [22,52,53]. Aberrant osteopontin levels in the endometrium have been linked to infertility [54].

Our target prediction analysis for miR-424-5p detected genes involved in numerous interleukins signalling pathways in the MS endometrium of fertile women, while the differential expression of a single miR-424-5p target gene, *SGK2*, was detected in the MS endometrium of RIF patients. Interleukins are a group of cytokines that regulate cell growth, differentiation, and they are particularly important in stimulating immune responses. The importance of immune responses in the pre- and peri-implantation periods in endometrial functions are widely acknowledged [4]. In order to provide a hospitable environment for the embryo, the balance between the maternal immune tolerance toward a semi-allogeneic implanting embryo and the protective anti-infectious mechanisms in the receptive phase uterus must be established [55]. Indeed, a recent study detected a range of interleukins to be down-regulated in infertile women with implantation failure in IVF [56]. SGK2, the potential target of miR-424-5p in RIF patients, is a protein kinase having powerful stimulating effect on K^+^ channels with a possible role in the regulation of epithelial transport and cell proliferation [57]. The importance of ion channels, including K^+^ in the regulation of endometrial receptivity has been summarised in a previous review [58]. However, to the best of our knowledge, *SGK2* has not been identified before in endometrial functions, and it could serve as an interesting target for future studies in women experiencing implantation failure.

Interaction analysis of our DE miRNAs and mRNAs identified the involvement of STAT3 and CDK5 signalling pathways in the development of RIF. CDK5 participates in a variety of cellular processes via the effects on angiogenesis, cell proliferation, cell adhesion, migration, and immune system (summarised in a recent review [59]).

Despite the differences in sample isolation protocols applied in the two cohorts, miR-30a-5p was elevated in the blood of RIF patients in both datasets. As miRNA levels in blood reflect its cellular composition, it is possible that the observed differences in miR-30a-5p levels between RIF patients and controls are derived from the alterations in the ratio of different cell types in blood samples. Although there are studies indicating that infertile women have altered levels of immune cells in blood, the results are highly conflicting [60], and therefore we cannot confirm nor rule out the possibility that miR-30a-5p alteration is due to variability in immune cell levels in RIF patients. Nevertheless, this miRNA could indirectly imply to the dysregulated physiological conditions resulting in implantation failure and serves as a potential minimally invasive biomarker in this regard. miR-30a-5p acts as a tumour suppressor by inhibiting cell proliferation and invasion [61,62,63]. In blood cells, miR-30a takes part in the regulation of erythrocyte maturation [64]. However, the precise mechanism that leads to the observed elevated levels in RIF patients’ blood in our study remains obscure.

As an additional finding in our study, we identified 18 novel miRNAs. Recently, a publication involving nearly 500 small RNA libraries from different mammalian primary cells analysed whether previously annotated and unannotated short RNA sequences serve as valid miRNAs [65]. Several sequences proposed as novel unannotated miRNAs in our study matched the genomic coordinates of proposed precursor sequences (including chr2_1900 and chr2_4401) and some met the multiple high-confidence criteria set for miRNAs in the aforementioned publication [65] (Table S5). This adds further confidence that the reported novel sequences in our study are genuine miRNAs. The most interesting finding to emerge from novel miRNA analysis was sequence chr2_4401 that showed higher expression levels in the MS vs. ES phase endometrium. Sequence alignment of chr2_4401 to several miR-200 family members demonstrates that their seed regions are identical. Therefore, we conclude that chr2_4401 plays similar important roles in MS endometrial functions as described above for the miR-200 family.

## 5. Conclusions

Our study approach, involving 230 samples, provides additional knowledge about the complex regulation of endometrial receptivity for successful embryo implantation. Our study results highlight the involvement of miR-30 and miR-200 family members in endometrial receptivity development, and miR-424-5p as dysregulated in RIF. The current study also identified several miRNAs not previously known to be involved in endometrial receptivity. We discovered several novel miRNAs, among which validated miRNA chr2_4401 is of most interest in MS endometrial functions. The results of our study provide new aspects of miRNA functions in MS endometrial processes in health and disease and provide means for further studies for identifying molecular biomarkers of fertility/infertility from endometrial and/or blood samples.

## Figures and Tables

**Table 1:** Novel miRNAs detected from endometrial and blood samples.

Novel miRNA Provisional ID	Sample Type	Consensus Mature Sequence	Endometrium			Blood		
			Fertile Women		Recurrent Implantation Failure (RIF) Patients	Fertile Women		RIF Patients
			Detected from Early Secretory (ES) Samples (*n* = 37)	Detected from Mid-Secretory (MS) Samples (*n* = 37)	Detected from MS Samples (*n* = 36)	Detected from ES Samples (*n* = 34)	Detected from MS Samples (*n* = 34)	Detected from MS Samples (*n* = 24)
chr2_1900	Endometrium	aucugaaauuugaaauggucc	-	3	4	-	-	-
chr2_4401	Endometrium	gaacacugaaguuaauggcug	1	26	29	-	-	-
chr2_2219	Blood	cugagaagacagucgaacuugac	-	-	-	11	7	5
chr3_7058	Endometrium, blood	aaaaguaaucgcggucuuugcc	-	2	-	3	4	5
chr3_1133	Endometrium, blood	caacaggccuugcucugcucacaga	1	-	2	2	-	2
chr3_2060	Endometrium	cacaggcaggagaccccacagc	7	2	1	-	-	-
chr4_6331	Endometrium	aacugggcauguuggaacuaagc	2	1	-	-	-	-
chr4_10018	Endometrium	aucagagaaacuuucuugaac	3	1	9	-	-	-
chr8_10314	Endometrium, blood	uuauccuccaguagacuaggga	18	8	14	3	2	4
chr8_8702	Endometrium, blood	acaccggggguuagagcuucaacc	2	-	-	-	1	2
chr10_9545	Endometrium, blood	aaaaguuauugcgguuuuugc	1	-	1	1	2	2
chr12_27523	Endometrium, blood	ucuggcuccuuucuaaucac	-	2	-	3	2	6
chr13_23821	Endometrium, blood	augugccuaguggcugcuguc	5	5	4	6	6	7
chr14_10307	Blood	ucugagcccuguucucccuagg	-	-	-	-	-	4
chr14_3458	Endometrium	aaaagucaucucgguucuugcc	3	1	3	-	-	-
chr15_26374	Endometrium	aaacguaauuguggauuuugc	2	1	3	-	-	-
chr16_22077	Endometrium	cugacugcccuggccuggccag	-	2	2	-	-	-
chr17_11615	Endometrium, blood	uaacucuuagaauccccaaag	1	-	-	18	16	10

## Supplementary Materials

The following are available online at http://www.mdpi.com/2073-4425/9/12/574/s1, Table S1: (a) miRNAs detected in EST endometrial samples. (b) miRNAs detected in ESP endometrial samples. (c) miRNAs detected in EST blood samples. (d) miRNAs detected in ESP blood samples. Table S2: (a) Differentially expressed miRNAs between MS and ES endometrial samples from fertile women. (b) Differentially expressed miRNAs from MS endometrial samples between RIF and fertile women. Table S3: (a) Predicted target genes for differentially expressed miRNAs in MS vs. ES phase endometrium from fertile women. (b) Predicted target genes for differentially expressed miRNAs in MS endometrium from fertile vs. infertile women with RIF. Table S4: (a) miR-424-5p target genes involved in different canonical pathways in MS phase endometrium. (b) Novel miRNA chr2_4401 target genes involved in different canonical pathways in MS phase endometrium. (c) Canonical pathways involved in MS endometrial functions (MS vs. ES endometrium from fertile women). (d) Canonical pathways involved in mid-secretory endometrial functions from infertile RIF women. Table S5: Novel miRNAs detected from endometrial and blood samples. Table S6: (a) All predicted targets for novel chr2_4401 miRNA (sequence: gaacacugaaguuaauggcug) according to miRDB. (b) Predicted targets for novel miRNA chr2_4401 down-regulated in MS endometrium.
